# Influenza B virus outbreak at a religious residential school for boys in Northern Bangladesh, 2011

**DOI:** 10.1111/irv.12430

**Published:** 2016-10-05

**Authors:** Farhana Haque, Katharine Sturm‐Ramirez, Nusrat Homaira, Emily Suzane Gurley, Md Jahangir Hossain, S. M. Murshid Hasan, Sukanta Chowdhury, Shamim Sarkar, Abdul Khaleque Md Dawlat Khan, Mustafizur Rahman, Mahmudur Rahman, Stephen P. Luby

**Affiliations:** ^1^ Programme on Emerging Infections (PEI)Infectious Diseases Division (IDD)icddr,bDhakaBangladesh; ^2^ Institute of EpidemiologyDisease Control and Research (IEDCR)DhakaBangladesh; ^3^ Centers for Disease Control and Prevention (CDC)AtlantaGeorgiaUSA

**Keywords:** Bangladesh, epidemic, influenza B virus, school dormitory

## Abstract

**Background:**

National media reported a febrile illness among dormitory residents of a boys' religious school. We investigated the outbreak to identify cause.

**Methods:**

Individuals with fever (>100°F) and cough or sore throat between 1 and 13 August 2011 were influenza‐like‐illness (ILI) case‐patients. We collected histories and specimens from hospitalized case‐patients and visited campus to explore environmental context.

**Results:**

All 28 case‐patients were dormitory residents including 27 hospitalizations. Accommodation space per resident was <0.8 square metres. Nasal and oropharyngeal swabs from 22 case‐patients were positive for influenza B virus using real‐time reverse transcription polymerase chain reaction (rRT‐PCR).

**Conclusions:**

Overcrowding likely facilitated transmission leading to this dormitory outbreak.

## 1 Introduction

On 9 August 2011, national mass media reported a febrile illness outbreak in a religious school for Muslim boys in Akkelpur subdistrict of Joypurhat District. Residential students developed fevers and headaches and were hospitalized in the government subdistrict hospital. As multiple countries have reported institutional respiratory outbreaks with high attack rates,^1^ a national, multidisciplinary, collaborative team from the Institute of Epidemiology, Disease Control and Research (IEDCR) of the Ministry of Health and icddr,b was formed to identify the cause and characterize the outbreak.

## 2 Methods

### 2.1 Epidemiological investigation

Local health officials visited the school, examined affected patients and established an acute respiratory illness outbreak. The collaborative team investigated during 10–13 August 2011. Based on preliminary information, the team suspected that this outbreak was caused by a respiratory virus, particularly influenza. The team identified and enlisted ILI case‐patients defined as individuals from the school and surrounding households with measured/reported fever (>100°F) and cough or sore throat from 1 to 13 August 2011. A pre‐tested, structured questionnaire was used to collect clinical and exposure histories from admitted case‐patients aged >10 years, but for children <10 years, the mother or a legal guardian was also asked to assist with responses. Team physicians examined case‐patients to note clinical features, reviewed medical records including chest radiographs to identify airspace opacity or consolidation, and assessed treatment plans to detect potential gaps in management.

Given that the majority of the non‐residential students resided in nearby households, the team conducted a door‐to‐door search in 50 households located within 15 minutes of walking distance from the school to identify ILI among non‐residential students. Local healthcare workers visited the school daily to follow‐up case‐patients' clinical progress and identify additional case‐patients. We also followed up case‐patients, family caregivers, school authorities and healthcare workers over telephone every alternate day until 21 August 2011 as influenza‐related complications usually occurred within 2 weeks of illness onset.^2^

### 2.2 Laboratory investigations

Trained medical technologists collected nasal and oropharyngeal swabs from hospitalized case‐patients, placed both swabs in a single vial containing viral transport media, and stored and transported at 4−8°C within 72 hours to icddr,b's Virology Laboratory. Both RNA and DNA extractions and real‐time reverse transcription polymerase chain reaction (rRT‐PCR) were performed following established methods to detect common respiratory viruses including subtypes of influenza A (H1, H3, H5), influenza B, respiratory syncytial virus, human parainfluenza virus 1, 2 and 3, human metapneumovirus and adenovirus.^3^

### 2.3 Anthropological investigations

In Bangladesh, influenza outbreaks were investigated using a multidisciplinary approach that combined synergies from quantitative and qualitative paradigms.^4^ Team anthropologists visited the campus, conducted in‐depth interviews and open discussions with admitted case‐patients, unaffected students, parents, teachers, community residents and healthcare providers to explore potential exposures including fluctuations in environmental temperature or rainfall, strategies for handling sick students in school and community perceptions and response.

### 2.4 Data analysis

We analysed the quantitative data to describe the outbreak in terms of person, place and time. We calculated ILI attack rate in the school by dividing the number of case‐patients with the total number of students and teachers. Similarly, we calculated the attack rate among dormitory residents. We reviewed the transcribed qualitative data to identify common themes, developed code list and summarized coded data according to objectives using principles of thematic content analysis.^5^

### 2.5 Ethical considerations

We sought verbal informed consent from the principal, all participants aged above 18 years, and local guardians of child participants, if available. Children at least 7 years of age were also asked to provide verbal assent. This investigation was approved by and undertaken jointly by icddr,b and the Ministry of Health and Family Welfare of the Government of Bangladesh.

## 3 Results

### 3.1 Description of the Islamic residential school

The residential (n=55) and non‐residential students (n=29), three teachers and principal in the school were all males. The school building with two rooms, each 6.7 m long and 3.3 m wide, served both as class rooms and as the dormitory after class.

### 3.2 Descriptive epidemiology

**Figure 1:**
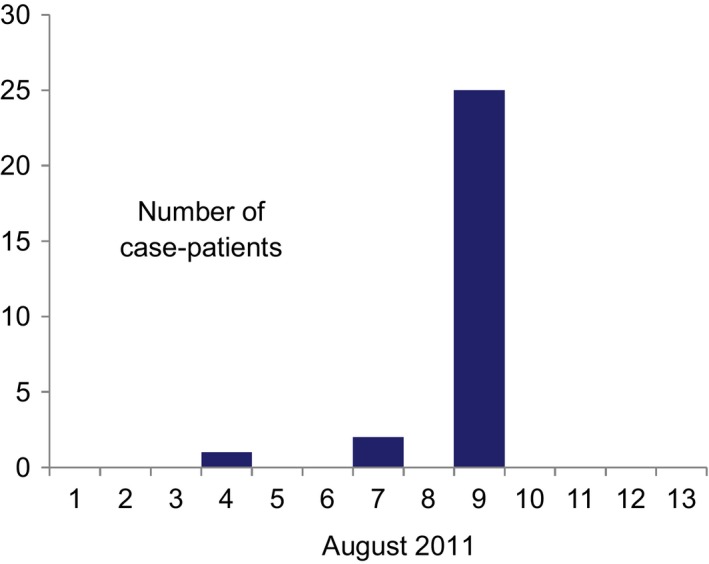
Date of onset of illness of case‐patients in the religious residential school for boys, Akkelpur subdistrict of Joypurhat District, Bangladesh, 1–13 August 2011 (n=28)

All 28 case‐patients were dormitory residents. Among these, 27 were hospitalized. The index case‐patient, a 16‐year‐old residential student, developed low‐grade fever, headache, mild cough and runny nose at 5:00 pm on 4 August. He was not hospitalized and recovered on 8 August 2011. Two students developed ILI on 7 August and 25 reported onset on 9 August 2011 (Fig. 1). The outbreak peaked during the first week of Ramadan, when Bangladeshi Muslims were fasting during daylight hours. The attack rate in school was 32% (28/88). Attack rates varied significantly between dormitory vs non‐dormitory students (28/59, 47% vs 0/29, 0%; *P*=0).

Given the lack of isolation facilities, hospitalized case‐patients were cohorted in a separate room away from other admitted patients. Their median age was 9 years (interquartile range, IQR: 8−11 years). Besides fever, predominant symptoms included cough (82%), headache (63%), runny nose (52%), sore throat (33%) and respiratory distress (11%) (Table 1). New case‐patients were neither identified from community search nor reported by local health team after 9 August.

**Table 1:** Clinical presentation, management and outcomes of influenza‐like illness (ILI) and laboratory confirmed influenza B case‐patients admitted to Akkelpur Upazila Health Complex, 11 August to 13 August 2011

	ILI case‐patients (%) n=27	Influenza B virus infected case‐patients (%) n=22
Clinical features		
Fever (>100^°^F) on admission	27 (100)	22 (100)
Cough	22 (82)	18 (82)
Headache	17 (63)	16 (73)
Runny nose/clear rhinorrhoea	14 (52)	12 (55)
Sore throat	9 (33)	9 (41)
Respiratory distress	3 (11)	3 (14)
Purulent sputum	4 (15)	4 (18)
Vomiting	4 (15)	4 (18)
Conjunctivitis	2 (7)	2 (9)
Abdominal pain	1 (4)	Nil
Localized crepitations	3 (11)	3 (14)
Expiratory rhonchi	10 (37)	8 (36)
Interstitial infiltration on chest radiograph (possible viral pneumonia)	3 (11)	2 (9)
Localized patchy opacities on chest radiograph (possible bacterial pneumonia)	3 (11)	3 (14)
Clinical management		
Antipyretic (acetaminophen)	27 (100)	22 (100)
Antibiotics	23 (85)	18 (82)
Oral bronchodilators	2 (7)	2 (9)
Antihistamine	25 (93)	21 (95)
Clinical outcomes or complications		
Death	0 (0)	0 (0)
Discharged following full recovery within 5 d	21 (78)	17 (77)
Discharged after partial recovery within 5 d	6 (22)	5 (23)

### 3.3 Laboratory findings

Collected swab samples from 22 of 27 hospitalized case‐patients interviewed that were tested on 18 August 2011 had detectable RNA for influenza B virus only.

### 3.4 Anthropological findings

#### 3.4.1 Interactions and habits of dormitory students

Students slept on the floor while a teacher slumbered on the bed in each room. The 44 square metres floor area provided 0.8 square metres of accommodation space per resident. Two small windows on adjacent walls in one room and a single window in the other allowed limited air exchange and restricted sunlight entry. During Ramadan, school ended 3 hours earlier at 1:00 pm and the fasting students mostly engaged in indoor rather than outdoor sports.

#### 3.4.2 Community perceptions and response

The healthcare manager, community residents and a teacher believed that the illness resulted from extreme heat followed by week‐long heavy rainfall before onset. Parents (6/10) believed that the nutrient‐poor diet (including rice, potatoes, lentils and boiled egg once a week) provided to the fasting students reduced their immunity.

After recognizing simultaneous illnesses in 25 students, the affected residents were immediately segregated into one dormitory room. The anxious media and the affected community demanded assistance. Health officials responded immediately and hospitalized all sick children.

## 4 Discussion

The sudden onset of ILI predominantly affecting dormitory student residents of a religious school signalled an outbreak; the detection of influenza B viral RNA in all collected samples confirmed it as the likely aetiologic agent. Influenza B is one of the most common circulating mammalian influenza viruses worldwide, frequently affecting school children.^6^ While the virus is known to circulate annually at low levels in Bangladesh,^7^ this outbreak where 47% of the residents reported ILI in less than a week, represented explosive transmission in the dormitory.

Less than one square metre of accommodation space per dormitory resident represented remarkable overcrowding. Changes in student behaviour during Ramadan and rainfall to stay indoors likely facilitated person‐to‐person transmission.^8^ The small windows limiting natural cross‐ventilation^9^ and restricted sunlight entry preventing viral inactivation by ultraviolet solar irradiation further increased scope of airborne transmission.^10^ Moreover, rainfall preceding outbreak onset likely increased environmental viral circulation.^11^ Children are more susceptible to influenza viruses than adults due to their limited lifetime exposures to annually circulating strains.^1^ The diurnal fasting could have further increased students' susceptibility to infection as intermittent calorie restriction in adults has been observed to suppress proinflammatory cytokine expression and lower circulating leucocyte counts.^12^

We identified this outbreak from monitoring mass media reports. The recognition of ILI in several dormitory residents simultaneously after awakening from sleep generated a sensational story that led to mass media reports instead of notification from the conventional public health channels. Even though the respiratory pathogen we identified was not a potential public health emergency, reporting of this small cluster in national media highlighted the potential utility of monitoring the mass media reports of outbreaks to promptly identify mild illness clusters.^13^ However, community panic along with media coverage heightened the demands from the public and the press for healthcare and real‐time information; this, in turn, likely led to excess hospitalizations and initiation of this field investigation led by national experts in the resource‐constrained Bangladesh.

## 5 Limitations

The detected number of case‐patients used to calculate the attack rate was likely an underestimate of the total number of infections, because our case definition likely failed to enrol case‐patients without fever. We also could not include the asymptomatic infections as this investigation of a mild, respiratory illness outbreak offered limited scope to objectively assess the apparently healthy residents for asymptomatic infections, which had been reported to be as high as 40% in prior outbreaks.^14^ Moreover, the limited community search may have failed to identify affected non‐residential students and/or their contacts.

## 6 Conclusion

Remarkable overcrowding likely facilitated viral transmission among susceptible children in the poorly ventilated dormitory. Increased tendency to stay indoors during Ramadan and rainfall further enhanced opportunities for transmission. As crowded dormitories are likely sites for rapid transmission of respiratory viruses, syndromic surveillance in selected dormitories may allow prompt detection of milder, respiratory disease clusters signalling potential public health emergencies.^15^

## Conflict of Interest

The authors do not have any commercial or other association that might pose a conflict of interest.

## Funding

This research activity was funded by the Centers for Disease Control and Prevention (CDC), Atlanta and the Government of the People's Republic of Bangladesh (GoB). icddr,b acknowledges with gratitude the commitment of the Centers for Disease Control and Prevention and the Government of Bangladesh to its research efforts. icddr,b is also grateful to the Governments of Bangladesh, Canada, Sweden and the UK for providing core/unrestricted support.

## Disclaimers

The interpretation and conclusions contained herein do not necessarily represent those of the Local Health Authority. The findings and conclusions in this report are those of the authors and do not necessarily represent the official position of the Centers for Disease Control and Prevention (CDC).

## Authors' Contributions

Farhana Haque, Stephen P Luby, M Jahangir Hossain and Mahmudur Rahman conceived the investigation. Farhana Haque, Katharine Sturm‐Ramirez, Emily S Gurley, Nusrat Homaira and M Jahangir Hossain designed the study protocol; Farhana Haque, AKM Dawlat Khan, SM Murshid Hasan, Sukanta Chowdhury and Shameem Sarkar carried out the epidemiological assessment. Farhana Haque, Mustafizur Rahman, AKM Dawlat Khan, SM Murshid Hasan, Sukanta Chowdhury, Shamim Sarkar and Mahmudur Rahman carried out the laboratory testing and analysis. Farhana Haque, Stephen P Luby, Katharine Sturm‐Ramirez and Emily S Gurley interpreted these data. Farhana Haque drafted the manuscript primarily; Stephen P Luby, Katharine Sturm‐Ramirez, Emily S Gurley and Nusrat Homaira critically revised the manuscript for intellectual content. All authors reviewed and approved the final manuscript.

## References

[1] Lessler J , Reich NG , Cummings DA , Nair HP , Jordan HT , Thompson N . Outbreak of 2009 pandemic influenza A (H1N1) at a New York City school. N Engl J Med. 2009;361:2628–2636. 20042754 10.1056/NEJMoa0906089

[2] Rothberg MB , Haessler SD , Brown RB . Complications of viral influenza. Am J Med. 2008;121:258–264. 18374680 10.1016/j.amjmed.2007.10.040 PMC7172971

[3] CDC protocol of realtime RTPCR for influenza A (H1N1) Pandemic (H1N1) 2009 guidance documents. Geneva, Switzerland: The WHO Collaborating Centre for influenza at CDC Atlanta; 2009: p. 1–7.

[4] Parveen S , Sultana R , Luby S , Gurley E . Anthropological approaches to outbreak investigations in Bangladesh In: Banwell C, Uligaszek S, Dixon J, eds. When Culture Impacts Health: Global Lessons From Asia and Australasia for Effective Health Interventions. London, UK: Academic Press; 2013: 215–224.

[5] Pope C , Ziebland S , Mays N . Qualitative research in health care. Analysing qualitative data. BMJ. 2000;320:114–116. 10625273 10.1136/bmj.320.7227.114 PMC1117368

[6] Mook P , Ellis J , Watson JM , et al. Public health implications of influenza B outbreaks in closed settings in the United Kingdom in the 2007/08 influenza season. Euro Surveill. 2008;13:pii: 18986. 18801323

[7] Zaman RU , Alamgir AS , Rahman M , et al. Influenza in outpatient ILI case‐patients in national hospital‐based surveillance, Bangladesh, 2007–2008. PLoS One. 2009;4:e8452. 20041114 10.1371/journal.pone.0008452 PMC2795194

[8] McNicholas A , Lennon D , Crampton P , Howden‐Chapman P . Overcrowding and infectious diseases–when will we learn the lessons of our past? N Z Med J. 2000;113:453–454. 11194749

[9] Escombe AR , Oeser CC , Gilman RH , et al. Natural ventilation for the prevention of airborne contagion. PLoS Med. 2007;4:e68. 17326709 10.1371/journal.pmed.0040068 PMC1808096

[10] Jensen MM . Inactivation of airborne viruses by ultraviolet irradiation. Appl Microbiol. 1964;12:418–420. 14215971 10.1128/am.12.5.418-420.1964 PMC1058147

[11] Cannell JJ , Zasloff M , Garland CF , Scragg R , Giovannucci E . On the epidemiology of influenza. Virol J. 2008;5:29. 18298852 10.1186/1743-422X-5-29 PMC2279112

[12] Faris MA , Kacimi S , Al‐Kurd RA , et al. Intermittent fasting during Ramadan attenuates proinflammatory cytokines and immune cells in healthy subjects. Nutr Res. 2012;32:947–955. 23244540 10.1016/j.nutres.2012.06.021

[13] Ao TT , Rahman M , Haque F , et al. Low‐Cost National media‐based surveillance system for Public Health Events, Bangladesh. Emerg Infect Dis. 2016;22:720–722. 26981877 10.3201/eid2204.150330 PMC4806969

[14] Chen SC , Liao CM . Probabilistic indoor transmission modeling for influenza (sub)type viruses. J Infect. 2009;60:26–35. 19818365 10.1016/j.jinf.2009.09.015

[15] Icddr, b . Clusters of severe respiratory infections identified through hospital‐based influenza surveillance, Bangladesh, 2009–2010. Health Sci Bull. 2010;8:12–18.

